# 593. PNPs in Burn Care: Enhancing Outcomes

**DOI:** 10.1093/jbcr/irag033.387

**Published:** 2026-04-06

**Authors:** Bénédicte Therrien-Hogue

**Affiliations:** Chu Sainte-Justine, Montréal-Ouest, Quebec

## Abstract

**Introduction:**

Managing pediatric burn injuries requires a specialized, interdisciplinary approach. The role of Pediatric Nurse Practitioners (PNPs) is often underestimated, yet their advanced clinical expertise and autonomy improve access to care, continuity of follow-up, and family experience. This presentation outlines PNP roles across the burn care continuum, highlights their impact on care quality and flow, and emphasizes their value to families and the care team.

**Methods:**

Based on clinical experience in a tertiary pediatric burn center, this descriptive presentation includes examples of PNP autonomy in managing complex wounds, adjusting therapies, and handling complications. It also showcases collaboration with surgeons, physiotherapists, psychologists, and others. PNPs play a key role in educating families and preparing them for discharge.

**Results:**

PNP integration improves accessibility and continuity of care. Their autonomy reduces decision-making delays, enhancing responsiveness. Their presence strengthens psychosocial support and promotes recognition of advanced nursing practice in specialized care.

**Conclusions:**

PNPs in pediatric burn units represent a major advancement in care quality and patient-family experience. This presentation aims to raise awareness of the need to fully recognize and deploy their role in specialized settings.

**Applicability of Research to Practice:**

Findings apply directly to pediatric burn care settings. PNPs’ autonomy enables timely, tailored decisions for complex needs, improving quality of patient care. Their role in education and coordination ensures smoother transitions home and fewer post-discharge complications. These practices are adaptable to other specialized contexts, reinforcing the importance of supporting advanced nursing practice across healthcare systems.

**Funding for the study:**

N/A.

